# Whole-genome analysis of monozygotic Brazilian twins discordant for type 1 narcolepsy: a case report

**DOI:** 10.1186/s12883-022-02921-w

**Published:** 2022-11-18

**Authors:** João H. C. Campos, Ana C. R. Aguilar, Fernando Antoneli, Giselle Truzzi, Marcelo R. S. Briones, Renata C. Ferreira, Fernando M. S. Coelho

**Affiliations:** 1 Center for Medical Bioinformatics, Escola Paulista de Medicina, UNIFESP, São Paulo, SP Brazil; 2 Department of Neurology and Neurosurgery, Escola Paulista de Medicina, UNIFESP, São Paulo, SP Brazil; 3 Department of Psychobiology, Escola Paulista de Medicina, UNIFESP, São Paulo, SP Brazil; 4 Epigene LLC, São Paulo, Brazil

**Keywords:** Narcolepsy type 1, Whole-genome analysis, HLA, Autoimmune disease, Discordant monozygotic twins, Diagnostics, Case report

## Abstract

**Background:**

Narcolepsy type 1 (NT1) is a rare and chronic neurological disease characterized by sudden sleep attacks, overwhelming daytime drowsiness, and cataplexy. When associated with a sudden loss of muscle tone (cataplexy) narcolepsy is classified as type 1, while the absence of cataplexy indicates type 2. Genetic, degenerative, and immunological hypotheses to explain the pathophysiology of NT1 are still a matter of debate. To contribute to the understanding of NT1 genetic basis, here we describe, for the first time, a whole genome analysis of a monozygotic twin pair discordant for NT1.

**Case presentation:**

We present the case of a pair of 17-year-old male, monozygotic twins discordant for NT1. The affected twin had Epworth Sleepiness Scale (ESS) of 20 (can range from 0 to 24), cataplexy, hypnagogic hallucinations, polysomnography without abnormalities, multiple sleep latency tests (MSLT) positive for narcolepsy, a mean sleep latency of 3 min, sleep-onset REM periods SOREMPs of 5, presence of allele HLA-DQB1*06:02, and Hypocretin-1 level of zero pg/mL (normal values are > 200 pg/mL). The other twin had no narcolepsy symptoms (ESS of 4), normal polysomnography, MSLT without abnormalities, presence of allele HLA-DQB1*06:02, and Hypocretin-1 level of 396,74 pg/mL. To describe the genetic background for the NT1 discordant manifestations in this case, we present the whole-genome analysis of this monozygotic twin pair. The whole-genome comparison revealed that both twins have identical NT1 pathogenic mutations in known genes, such as HLA-DQB1*06:02:01, HLA-DRB1*11:01:02/*15:03:01. The affected twin has the expected clinical manifestation while the unaffected twin has an unexpected phenotype. The unaffected twin has significantly more frameshift mutations as compared to the affected twin (108 versus 75) and mutations that affect stop codons (61 versus 5 in stop gain, 26 versus 2 in start lost).

**Conclusions:**

The differences observed in frameshift and stop codon mutations in the unaffected twin are consistent with loss-of-function effects and protective alleles, that are almost always associated with loss-of-function rare alleles. Also, overrepresentation analysis of genes containing variants with potential clinical relevance in the unaffected twin shows that most mutations are in genes related to immune regulation function, Golgi apparatus, MHC, and olfactory receptor. These observations support the hypothesis that NT1 has an immunological basis although protective mutations in non-HLA alleles might interfere with the expression of the NT1 phenotype and consequently, with the clinical manifestation of the disease.

**Supplementary Information:**

The online version contains supplementary material available at 10.1186/s12883-022-02921-w.

## Background

Narcolepsy is a rare neurological disease. Individuals affected by narcolepsy find it difficult to stay awake for long periods, causing serious disruptions in their daily routine. When associated with a sudden loss of muscle tone (cataplexy) narcolepsy is classified as type 1, while the absence of cataplexy indicates type 2 narcolepsy. Genetic, degenerative, and immunological hypotheses try to explain the pathophysiology of narcolepsy [1, 2]. Recently, several findings strongly suggest immunological causes for excessive daytime sleepiness, cataplexy, and other REM disturbances [3]. In the present study we compared the whole genomes of a monozygotic twin pair discordant for NT1 to provide data and analysis to contribute to the understanding of NT1 genetic basis.

In narcolepsy type 1 (NT1), the higher prevalence of allele HLA-DQB1*06:02 and Hypocretin (Hcrt) deficiency, supports the immunological theory for the cause of NT1 [4]. Genes that affect the CD4 + T cells antigen response influence the interaction of DQB1*06:02 and specific T cell receptors (TCRs) [5]. The autoantigen Trib2 is involved in human narcolepsy with anti-Tribble antibodies acting on hypocretin-producing neurons, leading to their disappearance and consequent Hcrt deficiency [6]. It has been proposed that a process of molecular mimicry must be involved given the observed increase of NT1 prevalence after the H1N1 influenza pandemic in 2009–2010 [7].

Excessive daytime sleepiness and cataplexy are keys for the diagnosis of narcolepsy, although many comorbidities are frequently observed in narcolepsy patients. In a study of a narcolepsy community sample, it has been observed a higher prevalence of obstructive sleep apnea, chronic low back pain, obesity, depression, and hyposmia [8]. Narcolepsy is related to other diseases such as Neuromyelitis Optica and thyroid disease with a possible overlap of autoimmune mechanisms [9]. Current findings on narcolepsy are the result of clinical, genetic, and laboratory studies of several research groups worldwide [1, 2, 10, 11]. Although discordant monozygotic twins for NT1 are extremely rare, a previous study characterized the HLA alleles in a twin pair discordant for NT1 and multiple sclerosis [12]. It must be noted that monozygotic twins are not genetically identical. They exhibit genetic differences mainly due to somatic mutations during development [13]. This case is unique because it is the first whole-genome analysis of monozygotic twins discordant for narcolepsy and there are no other narcolepsy cases in the family.

## Case presentation

We present a pair of 17-year-old male, monozygotic twins discordant for narcolepsy type I, that have been admitted in 2018 at the Daytime Excessive Sleepiness Ambulatory of the Federal University of Sao Paulo – UNIFESP. The twin with narcolepsy had excessive daytime sleepiness Epworth Sleepiness Scale (ESS) of 20 (can range from 0 to 24), cataplexy, hypnagogic hallucinations, polysomnography without abnormalities, multiple sleep latency tests (MSLT) positive for narcolepsy (a mean sleep latency of 3 min and sleep-onset REM periods SOREMPs of 5, presence of allele HLA-DQB1*06:02, and Hypocretin-1 level of zero pg/mL (normal values are > 200 pg/mL). He was treated with methylphenidate (20 mg/day) and imipramine (25 mg/day), with an improvement of symptomatology. The other twin had no narcolepsy symptoms (ESS of 4), normal polysomnography, MSLT without abnormalities, presence of allele HLA-DQB1*06:02, and Hypocretin-1 level of 396,74 pg/mL [14, 15]. There are no other narcolepsy cases and/or other autoimmune diseases (neurological or non- neurological) or any known infectious diseases in the immediate family or the expanded family respective to the twin pair here described. Both twins received a flu vaccine but it was not GSK. All methods are detailed in the Supplementary Material file “Methods”.

The genomes of both twins were sequenced with 30 × coverage. The genome assemblies were obtained for each individual using the reference human genome hg38 and the Genome Analysis Tool Kit (GATK) standards [16]. All nuclear chromosomes were assembled for the twin pair and the mitochondrial genomes, after assembled, were classified in the mitochondrial haplogroup L3e2b + 152 [17].

**Fig. 1:**
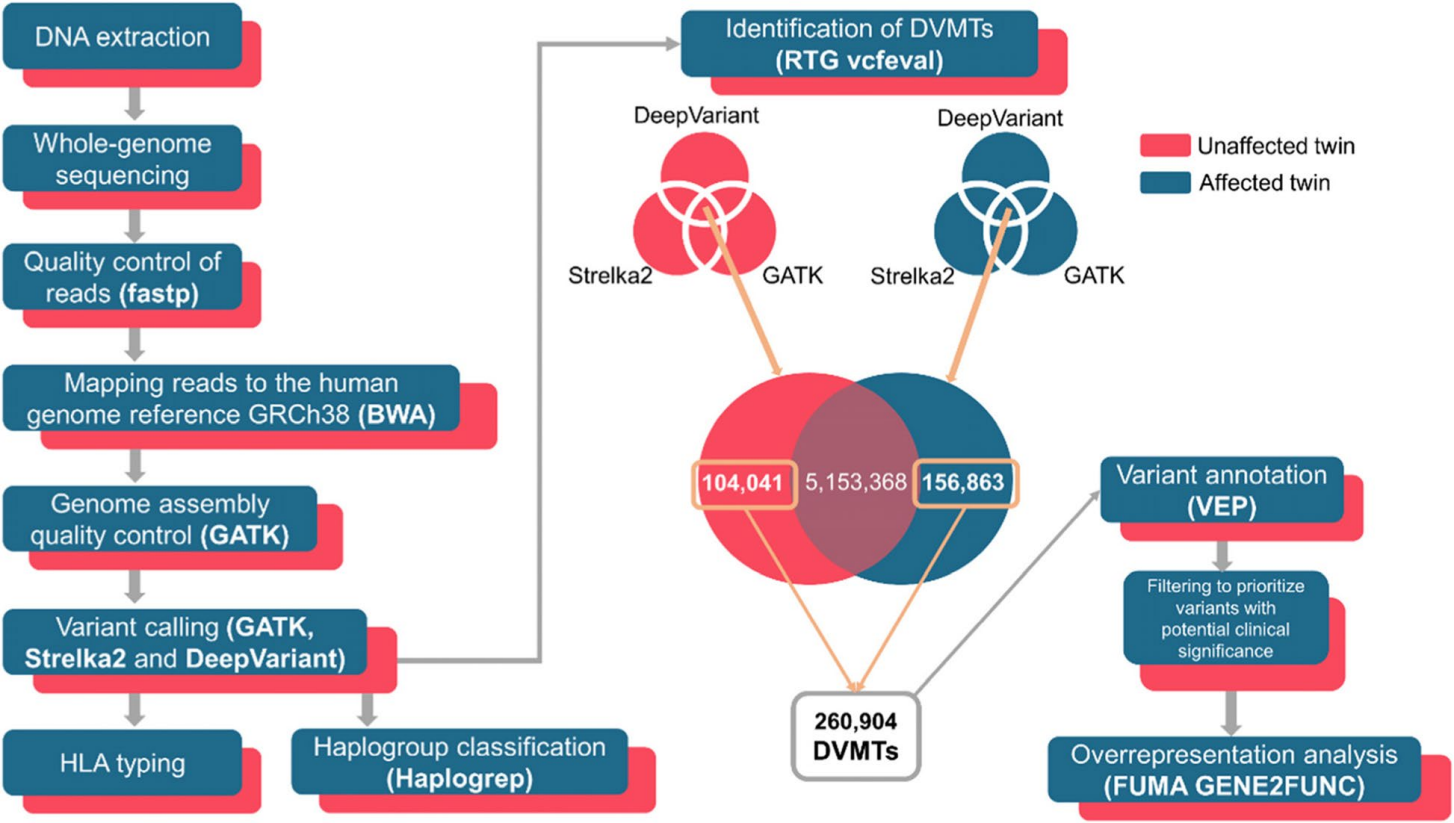
Genomic analysis pipeline of discordant twins for type 1 narcolepsy. Genomes of discordant twins for type 1 narcolepsy were sequenced, and the resulting reads processed for quality control. Good quality reads were then mapped against the human genome reference GRCh38. A new quality control over each assembly was carried out, and the variant calling step was conducted using three independent tools. After that, the consensus between variant call was determined for each twin: in the unaffected one, this resulted in a total of 5,257,409 variants, whereas in the affected twin, 5,310,227 variants. Unique variants were: 104,041 variants in the unaffected twin, and 156,863 variants in the affected twin, and classified as divergent variations in monozygotic twins (DVMTs). Only these 260,904 DVMTs were functionally annotated and filtered to prioritize SNVs with potential clinical relevance. Non-disruptive variants that might change protein effectiveness, or variants assumed to have high (disruptive) impact in the protein, probably causing protein truncation, loss of function or triggering nonsense mediated decay were included in a further enrichment analysis to provide insights about biological processes, cellular components and molecular functions of these DVMTs. Additionally, mitochondrial haplogroups, as well as the typing for the HLA alleles of each twin were determined. The parentheses highlight the tools used for each step

**Fig. 2:**
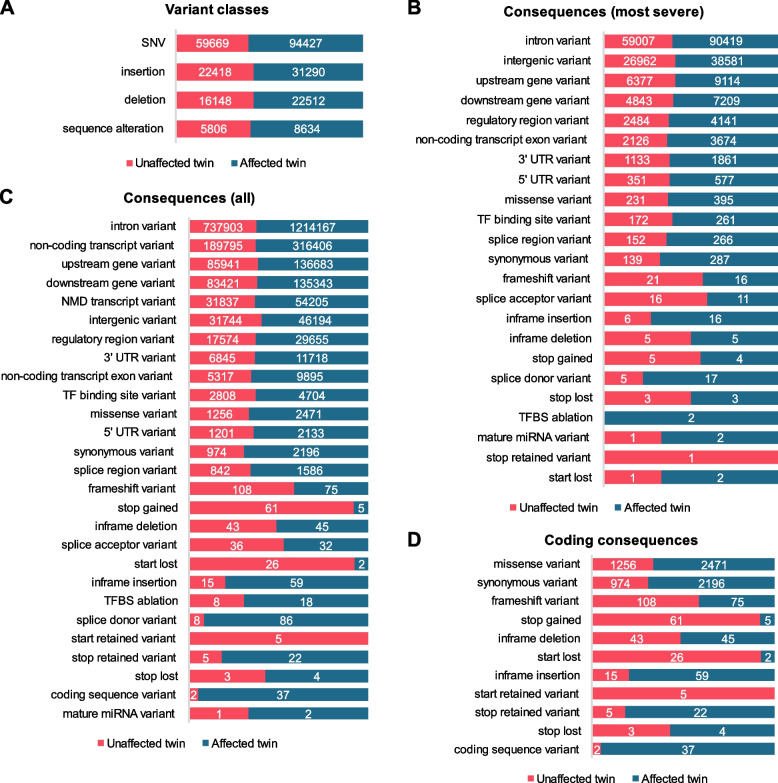
Annotation summary of divergent variations in monozygotic twins (DVMTs). All DVMTs were summarized for variant classes (**A**), most severe consequences (**B**), all consequences (**C**), and for coding consequences (**D**). Altogether, 104,041 DVMTs were found in the unaffected twin, and 156,863 in the affected twin (total = 206,904). In all summaries, the absolute count for each rating is indicated inside the bars (bars are proportional to the percentages found)

The strategy to find divergent variations in monozygotic twins (DVMTs) is outlined in Fig. 1, and a summary of the annotation of DVMTs found is represented in Fig. 2. The unaffected twin exhibited 5,257,409 variants in comparison with the human genome reference (GRCh38, Caucasian), whereas 5,310,227 variants were observed in affected twin in comparison to GRCh38. A total of 5,153,368 variants were common to twins and 104,041 variants were unique to the unaffected twin, and 156,863 were unique to the affected twin. Unique variants of each twin were classified as DVMTs, totalling 260,904 variants. This is 23-fold below the expected average 0.1% difference reported for non-twins genomes considering a diploid genome of 6 × 10^9^ bp (Figs. 1 and 2).

The HLA typing, obtained for a 1,000-fold coverage, shows that the twins have the same alleles for all HLA genes tested (Table 1). For genes *HLA-A*, *HLA-B* and *HLA-C* (MHC class I), the alleles are heterozygous; as for the MHC class II genes, only the *HLA-DQA1* and *HLA-DQB1* alleles are homozygous.

**Table 1:** HLA typing of the discordant monozygotic twin pair for NT1 with 1,000-fold deep coverage sequencing

HLA alleles	Unaffected	Affected
*HLA-A*	*30:02:01 / *34:02:01	*30:02:01 / *34:02:01
*HLA-B*	*14:02:01 / *53:01:01	*14:02:01 / *53:01:01
*HLA-C*	*04:01:01 / *08:02:01	*04:01:01 / *08:02:01
*HLA-DPA1*	*01:03:01 / *03:01:01	*01:03:01 / *03:01:01
*HLA-DPB1*	*02:01:02 / *04:02P	*02:01:02 / *04:02P
*HLA-DQA1*	*01:02:01 / *01:02:01	*01:02:01 / *01:02:01
*HLA-DQB1*	*06:02:01 / *06:02:01	*06:02:01 / *06:02:01
*HLA-DRB1*	*11:01:02 / *15:03:01	*11:01:02 / *15:03:01
*HLA-DRB3*	*02:02:01	*02:02:01
*HLA-DRB5*	*01:01:01	*01:01:01

In the unaffected twin, 244 loci have DVMTs with moderate impact (232 missense variants, 6 inframe insertions, 6 inframe deletions). These variants are distributed over 186 genes, most often in *MUC3A*, *MUC17,* and *HLA-B* (Supplementary Table 1). As for the variants with the impact of consequence classified as high, these are distributed in 51 loci (24 frameshift variants, 16 splice acceptor variants, 5 splice donor variants, 5 stop gained variants, 3 stop lost variants and 2 start lost variants – the number of consequences is greater because each variant can affect more than one transcript) occurring in 48 genes, mainly in *HGC6.3*, *HLA-A* and *HLA-B* (Supplementary Table 2).

A total of 68 variants were classified as deleterious by the SIFT predictor, mainly in genes *MUC17*, *MUC12,* and *GOLGA6L2* (Supplementary Table 3). The PolyPhen predictor, on the other hand, identified as damaging, 43 variants, in 37 genes (also mostly in *MUC17*, *MUC12*, and, additionally, in *FOXO3B*) (Supplementary Table 4). Damaging or deleterious variants identified with the two predictors add up to 29 and are located in 24 genes, occurring more frequently in *MUC12*, *FOXO3B,* and *MAP2K3* (Supplementary Table 5). 147 loci have variants in 37 genes associated with NT1 (described in the literature), notably in frequency in *HLA-DRB1*, *CACNA1C*, *HLA-B*, *NFATC2,* and *CCR3* (Supplementary Table 6). Among these genes, only *HLA-B* has variants with moderate or high impact of consequences (7 in total, where 5 are missense, and 2 are frameshift variants) (Supplementary Table 7). 103 variants were identified having clinical data in the ClinVar database, of which 13 have uncertain significance or conflicting interpretations of pathogenicity for 23 traits (Supplementary Table 8).

In the affected twin, 421 loci have DVMTs with moderate impact (399 missense, 16 inframe insertions, 8 inframe deletions), distributed in 323 genes, most often in *MUC4*, *LOC107986175*, *MUC20*, *MUC3A,* and *HLA-C* (Supplementary Table 9). As for the high impact variants, these are distributed in 53 loci (17 splice donor variants, 16 frameshift variants, 11 splice acceptor variants, 4 stop gained variants, 3 stop lost variants, and 2 start lost variants) occurring in 50 genes, mainly in *UBXN11*, *HLA-B,* and *PEX5* (Supplementary Table 10). 99 variants were classified as deleterious by the SIFT, mainly in the genes *MUC12*, *MUC4,* and *CLCN2* (Supplementary Table 11). PolyPhen identified 66 damaging variants in 55 genes (mostly in *MUC4*, *HLA-A,* and *HTR3D*) (Supplementary Table 12).

Damaging or deleterious variants identified with the two predictors total 46, arranged in 42 genes, and more frequently in *HLA-A*, *MUC20,* and *MUC4* (Supplementary Table 13). 177 loci have variants in 31 genes associated with NT1, notably in *HLA-B*, *CACNA1C*, *HLA-DRB1*, *SCP2,* and *NFATC2* (Supplementary Table 14). Among these, only *HLA-B* has variants with a consequence of moderate or high impact (6 in total, where 4 are missense and 2 are frameshift variants) (Supplementary Table 15). Finally, 162 variants were identified having data in ClinVar, of which 26 have uncertain significance or conflicting interpretations of pathogenicity for 31 traits (Supplementary Table 16).

**Fig. 3:**
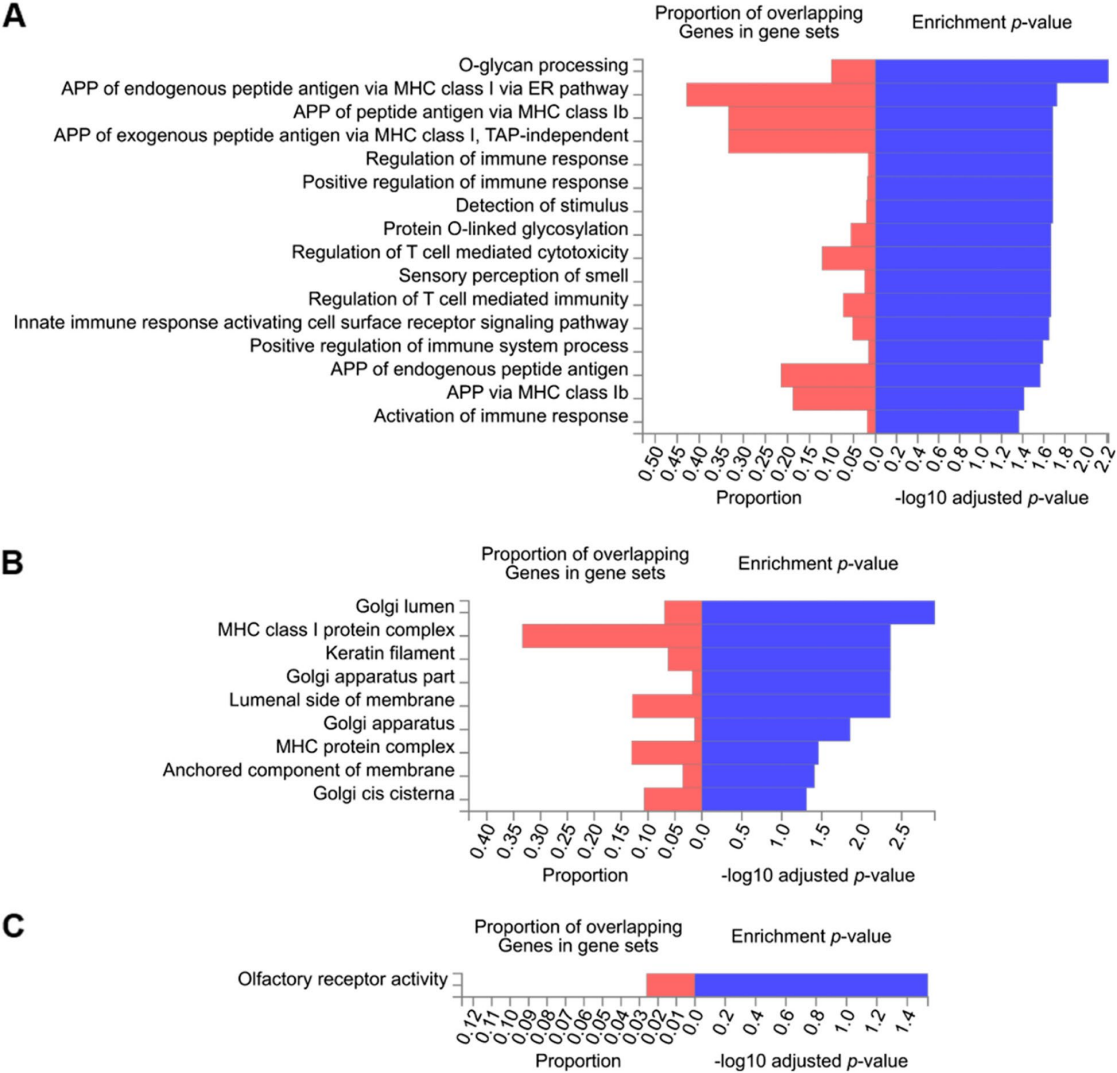
Overrepresentation analysis of genes containing variants with potential clinical relevance in the unaffected twin. Hypergeometric tests performed between the set of 227 genes that contains DVMTs of the unaffected twin, and the Gene Ontology (GO) gene sets for biological processes (BP), cellular components (CC) or molecular functions (MF) obtained from MsigDB database (https://www.gsea-msigdb.org/gsea/msigdb/), point to 16 overrepresented BPs (**A**), 9 CCs (**B**), and 1 overrepresented MF (**C**). Red bars indicate the proportion of genes tested (elements of the set of 227 genes) for each GO predefined gene set. Benjamini-Hochberg (FDR) multiple test correction method for enrichment testing was used, and gene sets with adjusted *p*-value < 0.05 were considered significative. The larger blue bars indicate that the test results are more likely to be non-random

Overrepresentation analysis of genes containing prioritized DVMTs of the unaffected twin showed 16 overrepresented in the Biological Properties (BPs) category, 9 in the Cellular Components (CCs) category, and 1 in the Molecular Function (MF) category (Fig. 3). BPs such as “antigen processing and presentation (APP) of endogenous peptide antigen via MHC class I via ER pathway” (GO:0,002,484), “APP of peptide antigen via MHC class Ib” (GO: 0,002,428), “APP of exogenous peptide antigen via MHC class I, TAP (transporter associated with antigen processing)-independent” (GO:0,002,480), “APP of endogenous peptide antigen” (GO:0,002,483), and “APP via MHC class Ib” (GO:0,002,475) have a proportion of overlapping genes in gene sets between ~ 18–45% (Fig. 3A). The highest proportion of overlapping genes in gene sets for CC (~ 33%) was for “MHC class I protein complex” (GO:0,042,612) (Fig. 3B), and “olfactory receptor activity” for MF (GO:0,004,984) (Fig. 3C).

The affected twin showed 55 overrepresented BPs, 18 CCs, and 10 MFs (Supplementary Figs. 1 and 2). The affected twin had the same overrepresented BPs, and CCs but with a higher proportion (~ 25–55% and ~ 45%, respectively) (Supplementary Figs. 1 and 2A). “Serotonin-gated cation-selective channel activity” (GO:0,022,850) was the most overrepresented MF in the affected twin (~ 60%) (Supplementary Fig. 2B).

Because olfactory alterations are associated with NT1 the twin pair was tested by using UPSIT [18]. The narcolepsy affected twin had severe hyposmia (30/40) while the unaffected twin had mild hyposmia (10/40).

## Discussion and conclusion

The whole-genome analysis of the discordant twins for NT1 revealed that the twin pair here considered has identical HLA alleles, particularly respective to allele HLA-DQB1*06:02:01 for which both are homozygous (Table 1). Because HLA-DQB1*06:02 is strongly associated with NT1, the clinical manifestations are as expected in the affected twin although the phenotype of the unaffected twin is unexpected. This suggests that genetic variants outside the HLA complex might be involved with the phenotype of the unaffected twin. Several variants in genes outside the HLA *loci* complex, such as *MUC17*, *MUC12*, *GOLGA6L2,* and *FOXO3B* were observed (Supplementary Tables 3 and 4). Also, the unaffected twin has significantly more frameshift mutations as compared to the affected twin (108 versus 75) and mutations that affect stop codons (61 versus 5 in stop gain, 26 versus 2 in start lost) (Fig. 2). The monozygotic twin pair belongs to the matrilineal haplogroup L3 (L3e2b + 152) which is of African/Middle Eastern ancestry [19]. This case, here reported, is unique because it is the first whole-genome analysis of monozygotic twins discordant for narcolepsy.

The overrepresentation analysis of genes containing variants with potential clinical relevance in the unaffected twin revealed that most mutations are in genes related to immune regulation function, Golgi apparatus, MHC, and olfactory receptor (Fig. 3). The observed mutations in olfactory genes are consistent with the NT1 UPSIT results. Although a previous study analysed the HLA locus of discordant monozygotic twins for narcolepsy type 1 and multiple sclerosis, their results are not identical to ours given the association with another disease and different ancestry types [12].

More importantly, most mutations found in the unaffected twin affect stop codons and/or cause frameshifts, which generally produce loss-of-function effects. The data here presented are fully consistent with several studies showing that protective alleles confer protection against disease by disrupting protein function, typically via loss-of-function (LoF) effects [20]. Although protective alleles are low-frequency or rare alleles the discovery of LoF protective alleles has stimulated the development of drugs that mimic gene LoF (or knockout) effects for several phenotypes. A successful example is the development of proprotein convertase subtilisin/kexin type 9 (PCSK9) inhibitors for the treatment of hypercholesterolemia [20, 21].

Besides loss-of-function effects, protective alleles in the unaffected twin might work as has been shown in the suppression of NF-kB signalling and HLA-C expression by *NLRP2* [22]. The detailed analysis of each individual mutation, and eventually the experimental proof of cause-consequence is a future development of this work. Another perspective is the study of distribution and frequencies of the non-HLA mutations, here characterized, in broader cohorts, particularly among patients belonging to mitochondrial haplogroup L3 (African/Middle Eastern ancestry) which are often underrepresented in genetic association studies [23].

The autoimmune mechanism is a combination of genetic predisposition and environmental triggers. It is often difficult to identify the factors responsible for the initiation of disease because most patients with autoimmune disease develop symptoms well after the abnormal immune reactions begin. A hypothesis for the genetic predisposition is that polymorphisms in various genes result in defective regulation or reduced threshold for lymphocyte activation. Cytokine and cytokine receptor genetic polymorphisms have been linked to many different autoimmune diseases. The unaffected twin has, for example, a stop-gain mutation in the MAP2K3 gene. The MAP2K3 protein is a dual specificity kinase, activated by cytokines and environmental stress in vivo. It catalyses the concomitant phosphorylation of a threonine and a tyrosine residue in the MAP kinase p38. MAPKs have been implicated as key regulators in the production of pro-inflammatory cytokines. A loss-of-function mutation may reduce inflammation and the damage of the cells responsible for hypocretin production which might explain in part the phenotype of the non-affected twin.

## Supplementary Information

**Additional file 1.** Methods.

**Additional file 2:** **Supplementary Table 1.**

**Additional file 3:** **Supplementary Table 2.**

**Additional file 4:** **Supplementary Table 3.**

**Additional file 5:** **Supplementary Table 4.**

**Additional file 6:** **Supplementary Table 5.**

**Additional file 7:** **Supplementary Table 6.**

**Additional file 8:** **Supplementary Table 7.**

**Additional file 9:** **Supplementary Table 8.**

**Additional file 10:** **Supplementary Table 9.**

**Additional file 11:** **Supplementary Table 10.**

**Additional file 12:** **Supplementary Table 11.**

**Additional file 13:** **Supplementary Table 12.**

**Additional file 14:** **Supplementary Table 13.**

**Additional file 15:** **Supplementary Table 14.**

**Additional file 16:** **Supplementary Table 15.**

**Additional file 17:** **Supplementary Table 16.**

**Additional file 18: Supplementary Figure 1.** Overrepresentation analysis of genes containing variants with potential clinical relevance in the affected twin – biological processes. Hypergeometric tests performed between the set of 362 genes that contains DVMTs of the affected twin, and the Gene Ontology (GO) gene sets for biological processes (BP) obtained from MsigDB database (https://www.gsea-msigdb.org/gsea/msigdb/), point to 55 overrepresented BPs. Red bars indicate the proportion of genes tested (elements of the set of 362 genes) for each GO predefined gene set. Benjamini-Hochberg (FDR) multiple test correction method for enrichment testing was used, and gene sets with adjusted *p*-value <0.05 were considered significative. The larger blue bars indicate that the test results are more likely to be non-random. **Supplementary Figure 2.** Overrepresentation analysis of genes containing variants with potential clinical relevance in the affected twin – cellular components or molecular functions. Hypergeometric tests performed between the set of 362 genes that contains DVMTs of the affected twin, and the Gene Ontology (GO) gene sets for cellular components (CC) or molecular functions (MF) obtained from MsigDB database (https://www.gsea-msigdb.org/gsea/msigdb/), point to 18 CCs and 10 overrepresented MFs. Red bars indicate the proportion of genes tested (elements of the set of 362 genes) for each GO predefined gene set. Benjamini-Hochberg (FDR) multiple test correction method for enrichment testing was used, and gene sets with adjusted *p*-value <0.05 were considered significative. The larger blue bars indicate that the test results are more likely to be non-random.

## Data Availability

All genome data presented in this report is deposited on GenBank SRA under the accession PRJNA807374 (to be released upon acceptance). Additional data requests should be directed to the corresponding authors.

## Abbreviations

NT1: Narcolepsy Type 1
HLA: Human Leukocyte Antigen
DVMTs: Divergent variations in monozygotic twins
ESS: Epworth Sleepiness Scale
MSLT: Multiple sleep latency tests
REM: Rapid eye movement
SOREMPs: Sleep-onset REM periods
UPSIT: The University of Pennsylvania Smell Identification Test
pg/mL: Picogram per milliliter
GO: Gene Ontology
GATK: Genome Analysis Toolkit
Hcrt: Hypocretin
