# Supplementary material for: Whole-genome analysis of monozygotic Brazilian twins discordant for type 1 narcolepsy: a case report
Source: BMC Neurol. 2022 Nov 18;22:439. doi: 10.1186/s12883-022-02921-w (PMC9673436; doi:10.1186/s12883-022-02921-w)
Supplement: Supplementary file 1 — Additional file 1. Methods. [file 12883_2022_2921_MOESM1_ESM.docx]

**Whole-genome analysis of monozygotic twins discordant for type 1 narcolepsy: a case report**

João H. C. Campos, Ana C. R. Aguilar, Fernando Antoneli, Giselle Truzzi, Marcelo R. S. Briones, Renata C. Ferreira*, Fernando M. S. Coelho*

**Methods**

**Whole-Genome Sequencing**

After signing the Informed Consent by the guardian and the Informed Consent Terms by both research participants, blood samples were collected. DNA samples were extracted using the QIAamp DNA Blood Mini Kit (Qiagen) according to the manufacturer's instructions and then sent for DNA sequencing at Centogene AG (Rostock, Mecklenburg-Vorpommern, Germany). Next-generation sequencing (NGS) of whole genomes was performed using Illumina HiSeq, with an approximately 30-fold average read depth coverage.

**Genome Assembly**

The pre-processing of FASTQ files which comprises quality control, adapter trimming, and quality filtering of raw data was done with fastp with recommended parameters [1]. All good reads were then aligned based on the human genome GRCh38 reference (https://www.ncbi.nlm.nih.gov/grc/human) using the Burrows-Wheeler transformation method, through BWA-MEM algorithm (bwa version 0.7.17) [2] After the initial mapping, resulting BAM files were submitted to the GATK best practices for rigid quality control of assemblies [3].

**Variant Identification**

Variant calling step was done using the GATK’s HaplotypeCaller algorithm, DeepVariant [4] or Illumina’s Strelka2 [5] tool. When HaplotypeCaller was used, our filtering criteria follows those adjusting parameters described elsewhere [6]. Strelka2 and DeepVariant were used with recommended default settings. The variant call consensus made by three tools (GATK, Strelka2, and DeepVariant) in each of the twins were obtained using RTG tools (version 3.10-5604f7a) (vcfeval algorithm) [7]. Unique variants were also found with vcfeval. When necessary, visual inspection of the assemblies was employed to increase the confidence in calls and to reduce the risk of false positives using IGV [8]. Finally, the annotation step of genetic variants was conducted using The Ensembl Variant Effect Predictor (VEP), API version 103 [9].

**Haplogroup Identification**

Mitochondrial haplogroup analysis was performed by comparison with PhyloTree [10] and Mitomap [11] haplogroup classification using HaploGrep2 [12].

**HLA Typing**

To identify HLA alleles, DNA samples were extracted using the QIAamp DNA Blood Mini Kit (Qiagen) according to the manufacturer's instructions and sent for DNA sequencing at *Instituto de Imunogenética* *IGEN/AFIP (*São Paulo, São Paulo State, Brazil). NGS of HLA genes were obtained using Illumina MiSeq, with a 1,000-fold average read depth of coverage. TypeStream™ Visual NGS Analysis Software (One Lambda, Canoga Park, California) was used for HLA typing.

**Variant Prioritization**

To prioritize DVMTs with potential clinical relevance, 3 main filtering criteria were established: (I) being a non-disruptive variant that might change protein effectiveness (impact of consequences predicted as "moderate"), (II) being variant assumed to have a high (disruptive) impact in the protein, probably causing protein truncation, loss of function or triggering nonsense-mediated decay (impact of consequences predicted as "high"), or (III) being considered damaging or deleterious variant by any of the 2 algorithms, SIFT or PolyPhen (included in VEP annotation output). We additionally evaluated DVMTs located in genes associated with NT1 (reported in the literature), or which have clinical data in ClinVar database https://www.ncbi.nlm.nih.gov/clinvar/.

**Overrepresentation Analysis**

Genes containing prioritized DVMTs were evaluated into hypergeometric tests using Gene Ontology (GO) data sets for biological processes (BP), cellular components (CC), or molecular functions (MF) with GENE2FUNC tool [13].

**Smell Identification Test**

The University of Pennsylvania Smell Identification Test (UPSIT) is a 40-item olfactory identification test, defining the olfaction acuity as “anosmia”, “severe hyposmia”, “moderate hyposmia”, “mild hyposmia”, and “normal” [14].

**References**

1. Chen S, Zhou Y, Chen Y, Gu J. fastp: an ultra-fast all-in-one FASTQ preprocessor. Bioinformatics. 2018;34:i884–90.

2. Li H. Aligning sequence reads, clone sequences and assembly contigs with BWA-MEM. arXiv:13033997 [q-bio]. 2013.

3. Van der Auwera GA, Carneiro MO, Hartl C, Poplin R, Del Angel G, Levy-Moonshine A, et al. From FastQ data to high confidence variant calls: the Genome Analysis Toolkit best practices pipeline. Curr Protoc Bioinformatics. 2013;43:11.10.1-11.10.33.

4. Poplin R, Chang P-C, Alexander D, Schwartz S, Colthurst T, Ku A, et al. A universal SNP and small-indel variant caller using deep neural networks. Nat Biotechnol. 2018;36:983–7.

5. Kim S, Scheffler K, Halpern AL, Bekritsky MA, Noh E, Källberg M, et al. Strelka2: fast and accurate calling of germline and somatic variants. Nat Methods. 2018;15:591–4.

6. Li H, Bloom JM, Farjoun Y, Fleharty M, Gauthier L, Neale B, et al. A synthetic-diploid benchmark for accurate variant-calling evaluation. Nat Methods. 2018;15:595–7.

7. Cleary JG, Braithwaite R, Gaastra K, Hilbush BS, Inglis S, Irvine SA, et al. Comparing Variant Call Files for Performance Benchmarking of Next-Generation Sequencing Variant Calling Pipelines. 2015.

8. Robinson JT, Thorvaldsdóttir H, Wenger AM, Zehir A, Mesirov JP. Variant Review with the Integrative Genomics Viewer. Cancer Res. 2017;77:e31–4.

9. McLaren W, Gil L, Hunt SE, Riat HS, Ritchie GRS, Thormann A, et al. The Ensembl Variant Effect Predictor. Genome Biology. 2016;17:122.

10. van Oven M, Kayser M. Updated comprehensive phylogenetic tree of global human mitochondrial DNA variation. Human Mutation. 2009;30:E386–94.

11. Lott MT, Leipzig JN, Derbeneva O, Xie HM, Chalkia D, Sarmady M, et al. mtDNA Variation and Analysis Using Mitomap and Mitomaster. Curr Protoc Bioinformatics. 2013;44:1.23.1-26.

12. Weissensteiner H, Pacher D, Kloss-Brandstätter A, Forer L, Specht G, Bandelt H-J, et al. HaploGrep 2: mitochondrial haplogroup classification in the era of high-throughput sequencing. Nucleic Acids Res. 2016;44:W58–63.

13. Watanabe K, Taskesen E, van Bochoven A, Posthuma D. Functional mapping and annotation of genetic associations with FUMA. Nat Commun. 2017;8:1826.

14. Doty RL, Shaman P, Kimmelman CP, Dann MS. University of pennsylvania smell identification test: A rapid quantitative olfactory function test for the clinic. The Laryngoscope. 1984;94:176–8.
