# Supplementary material for: Whole-genome analysis of monozygotic Brazilian twins discordant for type 1 narcolepsy: a case report
Source: BMC Neurol. 2022 Nov 18;22:439. doi: 10.1186/s12883-022-02921-w (PMC9673436; doi:10.1186/s12883-022-02921-w)
Supplement: Supplementary file 18 — Additional file 18: Supplementary Figure 1. Overrepresentation analysis of genes containing variants with potential clinical relevance in the affected twin – biological processes. Hypergeometric tests performed between the set of 362 genes that contains DVMTs of the affected twin, and the Gene Ontology (GO) gene sets for biological processes (BP) obtained from MsigDB database (https://www.gsea-msigdb.org/gsea/msigdb/), point to 55 overrepresented BPs. Red bars indicate the proportion of genes tested (elements of the set of 362 genes) for each GO predefined gene set. Benjamini-Hochberg (FDR) multiple test correction method for enrichment testing was used, and gene sets with adjusted p-value <0.05 were considered significative. The larger blue bars indicate that the test results are more likely to be non-random. Supplementary Figure 2. Overrepresentation analysis of genes containing variants with potential clinical relevance in the affected twin – cellular components or molecular functions. Hypergeometric tests performed between the set of 362 genes that contains DVMTs of the affected twin, and the Gene Ontology (GO) gene sets for cellular components (CC) or molecular functions (MF) obtained from MsigDB database (https://www.gsea-msigdb.org/gsea/msigdb/), point to 18 CCs and 10 overrepresented MFs. Red bars indicate the proportion of genes tested (elements of the set of 362 genes) for each GO predefined gene set. Benjamini-Hochberg (FDR) multiple test correction method for enrichment testing was used, and gene sets with adjusted p-value <0.05 were considered significative. The larger blue bars indicate that the test results are more likely to be non-random. [file 12883_2022_2921_MOESM18_ESM.pdf]

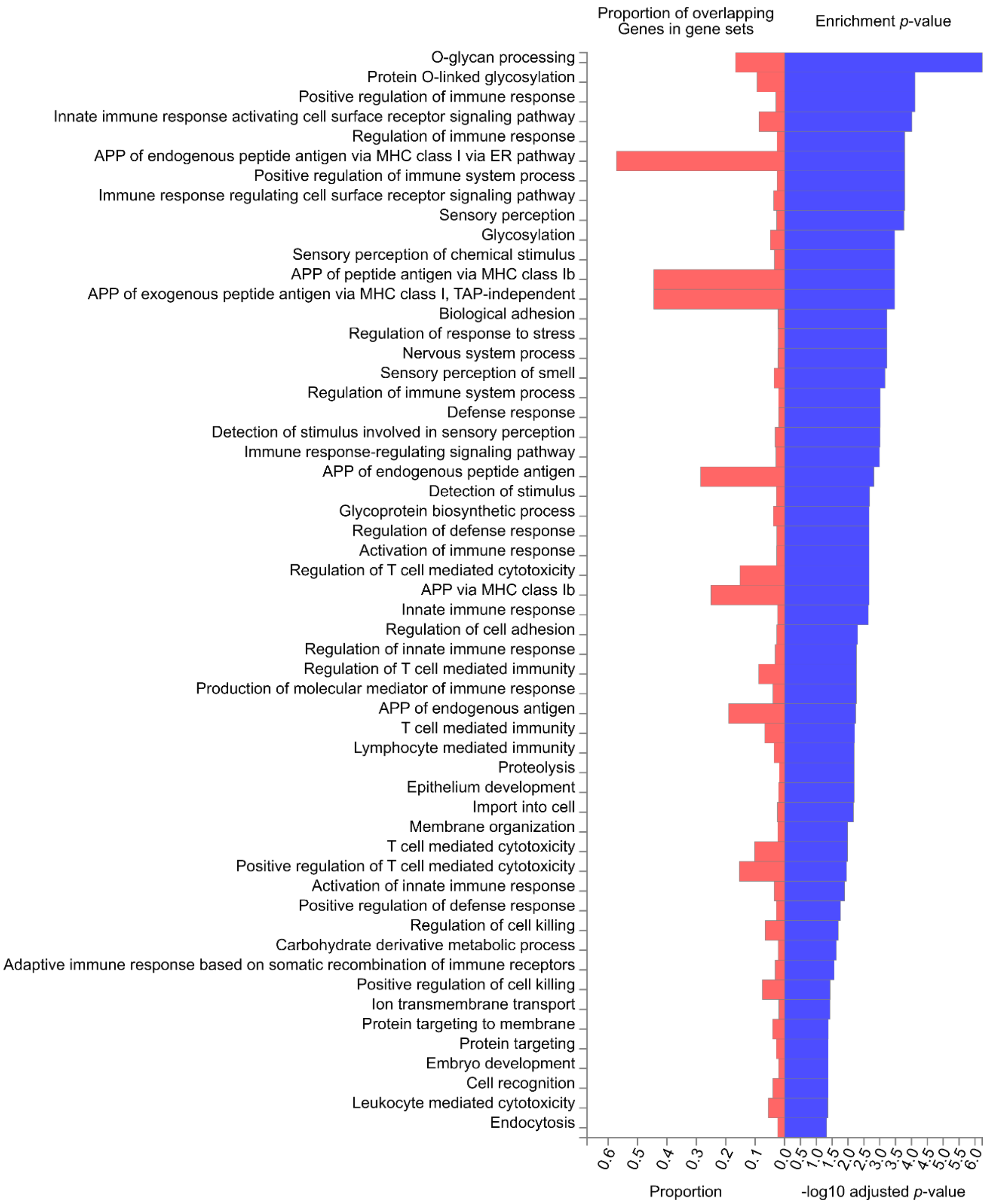

Supplementary Figure 1. Overrepresentation analysis of genes containing variants with potential clinical relevance in the affected twin – biological processes. Hypergeometric tests performed between the set of 362 genes that contains DVMTs of the affected twin, and the Gene Ontology (GO) gene sets for biological processes (BP) obtained from MsigDB database (https://www.gsea-msigdb.org/gsea/msigdb/), point to 55 overrepresented BPs. Red bars indicate the proportion of genes tested (elements of the set of 362 genes) for each GO predefined gene set. Benjamini-Hochberg (FDR) multiple test correction method for enrichment testing was used, and gene sets with adjusted *p*-value <0.05 were considered significative. The larger blue bars indicate that the test results are more likely to be non-random.

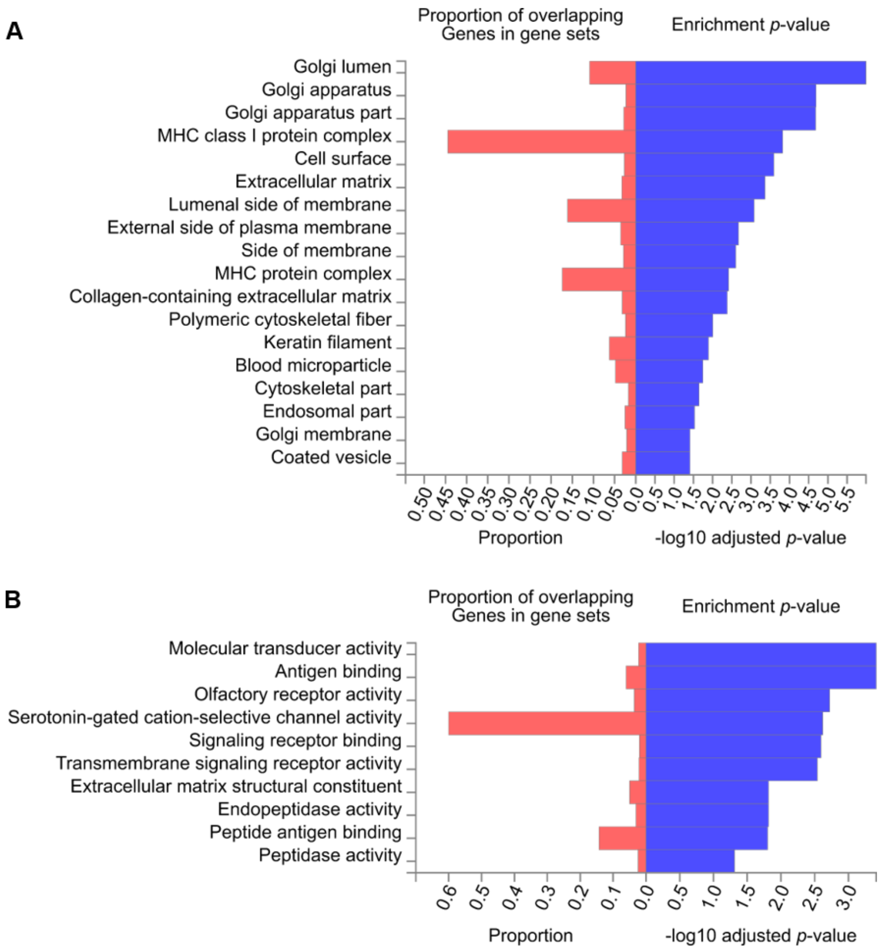

Supplementary Figure 2. Overrepresentation analysis of genes containing variants with potential clinical relevance in the affected twin – cellular components or molecular functions. Hypergeometric tests performed between the set of 362 genes that contains DVMTs of the affected twin, and the Gene Ontology (GO) gene sets for cellular components (CC) or molecular functions (MF) obtained from MsigDB database (https://www.gsea-msigdb.org/gsea/msigdb/), point to 18 CCs and 10 overrepresented MFs. Red bars indicate the proportion of genes tested (elements of the set of 362 genes) for each GO predefined gene set. Benjamini-Hochberg (FDR) multiple test correction method for enrichment testing was used, and gene sets with adjusted *p*-value <0.05 were considered significative. The larger blue bars indicate that the test results are more likely to be non-random.
